# Freeze‐Drying *Chlorella vulgaris* by Using Aquafaba, Deactivated Yeast, Inulin and Maltodextrin

**DOI:** 10.1002/fsn3.71897

**Published:** 2026-05-28

**Authors:** Faruk Tamtürk, Başak Gürbüz, Sevim Dalabasmaz, Yaşar Durmaz, Nevzat Konar, Ömer Said Toker, Atefeh Karimidastjerd

**Affiliations:** ^1^ Food Engineering Department, Faculty of Chemical and Metallurgical Engineering Yıldız Technical University İstanbul Turkiye; ^2^ Biorld R&D Center Istanbul Turkiye; ^3^ Tayas Food R&D Center Kocaeli Turkiye; ^4^ Food Chemistry, Department of Chemistry and Pharmacy Friedrich‐Alexander‐Universität Erlangen‐Nürnberg (FAU) Erlangen Germany; ^5^ Aquaculture Department, Faculty of Fisheries Ege University Izmir Turkey; ^6^ Dairy Technology Department, Agriculture Faculty Ankara University Ankara Turkey; ^7^ Institute of Food Safety Ankara University Ankara Turkey

**Keywords:** *Chlorella vulgaris*, encapsulation efficiency, microalgae preservation, microencapsulation

## Abstract

The effects of novel wall materials for *Chlorella vulgaris* were reported using spray‐drying in our previous study. In this study, we extend this work by applying freeze‐drying, another major and widely used encapsulation technique, and encapsulate *C. vulgaris* biomass using this method. The experimental design was prepared using the Simplex‐Lattice Mixture Design method. The independent variables were combinations of various wall materials such as maltodextrin (18–20 DE), inulin (DP < 10), aquafaba, and deactivated yeast as spray‐dried *Saccharomyces cerevisiae* cells (25.0%–75.0%, w/w, in dm) and *C. vulgaris* biomass (25.00%–100.0%, w/w, in dm). The amounts of pigments, crude protein, physicochemical and color properties, wettability, hygroscopicity, and drying and encapsulation efficiency of the samples were determined. Significant models (*p* < 0.05) for moisture (1.30–3.36 g/100 g), total carotenoid (0.36–1.62 mg/g), total chlorophylls (8.51–29.8 mg/g), and crude protein (6.40–43.8 g/100 g) contents were obtained. Furthermore, it was found that the size and coalescence trends of the samples were influenced by the maltodextrin ratio used. Based on the results of this study, innovative materials such as deactivated yeast and aquafaba have significant potential for use in microalgae encapsulation and drying.

## Graphical abstract

This study investigates the freeze‐drying efficiency of *Chlorella vulgaris* cultivated in a photobioreactor, utilizing novel and traditional carrier agents including aquafaba, deactivated yeast, inulin, and maltodextrin. The findings highlight the comparative effects of these matrices on the structural integrity and physical properties of the microalgae powder, offering sustainable processing approaches for functional food applications.

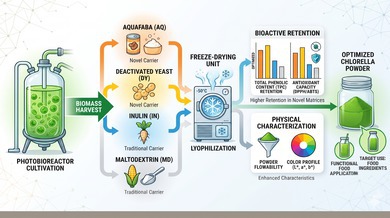

## 1 Introduction

Microalgae have gained significant attention as sustainable and nutrient‐dense food sources, offering advantages over traditional terrestrial agriculture due to their minimal land requirements, independence from soil and irrigation, and high productivity. As a result, replacing or complementing conventional agricultural products with microalgae can reduce pressure on land and water resources while supporting climate‐friendly food production systems (Greene et al. 2022). Among edible microalgae, *Chlorella vulgaris* is one of the most widely cultivated species owing to its rich protein profile (42%–58%), essential amino acids, minerals, chlorophylls, carotenoids, and polysaccharides, which together make it a promising ingredient for functional food applications (Vieira et al. 2020). Beyond its nutritional value, *C. vulgaris* contains bioactive compounds with anticancer, antioxidant, anti‐inflammatory, antimicrobial, and immunomodulatory functions (Vieira et al. 2020; Rua et al. 2020). Its high chlorophyll and lutein contents also make it a potential natural green colorant, despite the instability of these pigments when exposed to heat, oxygen, pH changes, and light (Kulkarni and Nikolov 2018; Sarkar et al. 2020). Encapsulation is therefore considered an effective strategy to improve pigment stability and protect sensitive bioactive compounds during processing and storage (Agustina et al. 2021). *C. vulgaris* biomass contains significant amounts of chlorophylls, carotenoids, proteins, carbohydrates, lipids, and B vitamins (B1, B2, B6, and B12) and is therefore widely used. The crude protein concentration in this algal biomass has been previously reported to range from 21% to 60% of dry weight (w/w), with this variation depending on factors such as cultivation and harvesting conditions, as shown by previous research (Konar et al. 2022; Stramarkou et al. 2017; Amin et al. 2021). The protein nutritional quality of *C. vulgaris* conforms to the standard profile for human nutrition recommended by the World Health Organization (WHO) and the Food and Agriculture Organization (FAO) (Safi et al. 2014). The biomass of *C. vulgaris* is an important source of algal protein (Konar et al. 2022). The chemical composition of microalgal biomass depends on factors such as the specific species and strain, growing conditions, and various other factors.

Although microalgae are commonly marketed as powders, tablets, or capsules, their incorporation into food matrices is rapidly increasing in response to demand for functional and plant‐based products (Lafarga 2019). To support this growth, improving the stability, sensory properties, and functional behavior of microalgae ingredients through encapsulation technologies is essential. Freeze‐drying is a widely used method for encapsulating thermolabile compounds and has shown strong potential for preserving microalgal pigments and bioactives (Vieira et al. 2020).

Alongside processing techniques, the selection of appropriate wall materials plays a critical role in determining encapsulation yield, efficiency, stability, and physicochemical quality. In this context, innovative encapsulation agents with both technological and functional properties are being explored. Aquafaba—a legume‐derived extract—is valued for its foaming, emulsifying, binding, and thickening capabilities (Hedayati et al. 2022). Deactivated *Saccharomyces cerevisiae* yeast cells serve as biodegradable, GRAS‐status microcapsules capable of protecting sensitive compounds due to their robust cell wall structure (Mokhtari et al. 2017; Shi et al. 2010; Dadkhodazade et al. 2021). Inulin, a natural dietary fiber, offers stabilizing, emulsifying, and prebiotic properties and has previously been used as a wall material in pigment encapsulation (Šeregelj et al. 2021; Oliveira et al. 2022). Maltodextrin remains one of the most widely used encapsulants because of its solubility, low viscosity, and neutral sensory profile, although it often requires combination with other agents due to its limited emulsifying capacity (Todorović et al. 2022). The aim of this study is to determine the optimum formulation of wall materials including deactivated baker's yeast (spray‐dried *S. cerevisiae* cells), aquafaba, inulin, and maltodextrin for *C. vulgaris* freeze‐drying by using Simplex‐Lattice Mixture Design. Innovative encapsulation agents such as aquafaba and deactivated yeast with both functional and technological properties were selected in the study. Encapsulation efficiency, encapsulation yield, and final product moisture were investigated as a response to the determination of optimum wall material combination.

## 2 Materials and Methods

### 2.1 *Chlorella vulgaris* Strain and Encapsulating Agents

*Chlorella vulgaris* strain (CCAP 211/52) was cultivated using the method and equipment described by Tamtürk et al. (2023). Maltodextrin (18–20 DE; Cargill, Istanbul, Turkey), inulin (degree of polymerization < 10; Tito, Istanbul, Turkey), aquafaba powder (Döhler Food, Karaman, Turkey) and deactivated yeast in the form of spray‐dried *S. cerevisiae* cells (Pak Maya, Istanbul, Turkey) were used as encapsulating agents. Feed solutions included 10% (m/m) dry matter and kept at −80°C until freeze‐drying.

### 2.2 Study Model

The experimental design was a Simplex‐Lattice Mixture Design carried out using statistical software for experimental design and optimization called Design‐Expert (Version 13.0.8.0; State‐Ease Minneapolis, MN, USA). All of the initial feeding solutions were prepared for the optimization procedure (Table 1). In each experiment, encapsulation yield and encapsulation efficiency were evaluated as the response variables. Based on preliminary trials, the lower and upper limits for the formulation components were defined as follows: *X*_1_ (25%–100%) and *X*_2_, *X*_3_, *X*_4_, and *X*_5_ (0%–75%), whereas ensuring that the total proportion of all ingredients always summed to 100%. The responses or the dependent variables evaluated for each run were encapsulation yield, encapsulation efficiencies of total chlorophylls and total carotenoids. The Scheffé model was fitted using polynomial equations to correlate the response variables (*Y*) to the independent variables (*X*). Equation (1) is as follows (Tamtürk et al. 2023):

(1) $$Y=\beta_{i}X_{i}+\beta_{\mathrm{ij}}X_{i}X_{j}\;1\leq i\leq q\;1\leq i<j\leq q$$

**TABLE 1:** Encapsulation optimization parameters of *Chlorella vulgaris* .

Run	Algae biomass (%)	Deactivated bakery yeast (%)	Aquafaba (%)	Inulin (%)	Maltodextrin (%)	EY (%)	Chlorophyll‐a EE_1_ (%)	Total carotenoids EE_2_ (%)
1	100	0.00	0.00	0.00	0.00	95.5	62.7	46.7
2	25.0	75.0	0.00	0.00	0.00	96.4	97.8	55.5
3	25.0	0.00	75.0	0.00	0.00	99.9	97.7	60.6
4	25.0	0.00	0.00	75.0	0.00	98.2	96.4	70.1
5	25.0	0.00	0.00	0.00	75.0	98.4	81.4	41.1
6	62.5	37.5	0.00	0.00	0.00	97.7	90.5	68.7
7	62.5	0.00	37.5	0.00	0.00	98.1	76.1	73.3
8	62.5	0.00	0.00	37.5	0.00	96.1	60.6	64.2
9	62.5	0.00	0.00	0.00	37.5	98.7	60.5	59.9
10	25.0	37.5	37.5	0.00	0.00	98.9	94.9	95.2
11	25.0	37.5	0.00	37.5	0.00	97.5	88.7	77.3
12	25.0	37.5	0.00	0.00	37.5	96.4	88.8	80.5
13	25.0	0.00	37.5	37.5	0.00	98.5	91.7	95.6
14	25.0	0.00	37.5	0.00	37.5	97.9	77.4	65.5
15	25.0	0.00	0.00	37.5	37.5	97.6	95.7	93.4
16	70.0	7.50	7.50	7.50	7.50	98.5	63.2	62.4
17	32.5	45.0	7.50	7.50	7.50	99.4	92.4	86.4
18	32.5	7.50	45.0	7.50	7.50	98.1	82.1	74.0
19	32.5	7.50	7.50	45.0	7.50	98.9	78.2	62.4
20	32.5	7.50	7.50	7.50	45.0	95.7	68.2	48.9
21	40.0	15.0	15.0	15.0	15.0	97.7	77.5	51.5
22	100.0	0.00	0.00	0.00	0.00	95.2	52.9	46.3
23	25.0	75.0	0.00	0.00	0.00	97.2	98.9	59.0
24	25.0	0.00	75.0	0.00	0.00	98.1	96.2	68.4
25	25.0	0.00	0.00	75.0	0.00	97.9	94.6	81.2
26	25.0	0.00	0.00	0.00	75.0	98.4	71.5	48.6
Model						Special cubic	Quadratic	Special cubic
*p* value						0.0341*	< 0.0001*	0.0034*
Lack of fit *F* value						0.8398**	1.18**	8.26**
Model *R* ^2^						0.9353	0.9511	0.9723

*Note:* Mean ± standard deviation. Analyses were performed in triplicate.

Abbreviations: EE, encapsulation efficiency; EY, encapsulation yield.

* Significant.

** Not significant. *p* < 0.05.

In a mixture experiment, it is not the volume of the actual amount of each ingredient that matters, but rather its proportion in relation to other ingredients. The sum of all the ingredients is a constant total *T*, which is equal to 100% or 1 unless any constant mixture factors are present.

Therefore, if *X*_1_, *X*_2_, *X*_3_, … *X*_*i*_ denotes the proportions of components of a mixture, then:

(2) $$\mathrm{Sq}-1:\sum_{i=1}^{q}X_{i}=1,0\leq X_{i}\leq 1,i=1,2,\ldots ,q$$

(3) $$X_{1}+X_{2}+X_{3}+X_{4}+X_{5}=100\%\left(i.e.,1\right)$$

where *X*_1_, *X*_2_, *X*_3_, *X*_4_, and *X*_5_ were ratios of *C. vulgaris* biomass, deactivated yeast, aquafaba, inulin, and maltodextrin in dry mass of feed solution, respectively.

### 2.3 Encapsulation of *C. vulgaris* Using a Freeze Dryer

The solutions prepared at a concentration of 20% (w/v) were frozen at −80°C. They were then dried in a freeze dryer (Epsilon, Christ, Osterode, Germany) at 0.05 mbar vacuum for 48 h (Gani et al. 2018).

### 2.4 Encapsulation Efficiency and Yield

Encapsulation efficiency of total chlorophylls (EE_1_, %) and total carotenoids (EE_2_, %) was determined by using Equation (4). Encapsulation yield (EY, %) was determined by using Equation (5) (Tamtürk et al. 2023; Konar et al. 2022; Nunes and Mercadante 2007).

(4) $$\mathrm{EE}\;\left(\%\right)=100\times \frac{\left[\text{Pigment amount of feed solution}\;\left(\text{in}\;\mathrm{dm}\right)-\text{amount of encapsulated pigment}\;\left(\text{in}\;\mathrm{dm}\right)\right]}{\text{Pigment amount of feed solution}\;\left(\text{in}\;\mathrm{dm}\right)}$$

(5) $$\mathrm{EY}\;\left(\%\right)=100\times \frac{\left[\text{Total mass of feed solution}\;\left(\text{in}\;\mathrm{dm}\right)-\text{encapsulated mass}\;\left(\text{in}\;\mathrm{dm}\right)\right]}{\text{Total mass of feed solution}\;\left(\text{in}\;\mathrm{dm}\right)}$$

### 2.5 Pigment Contents

To determine the total chlorophylls and total carotenoid amounts in the samples, a UV–VIS spectrophotometer (UV‐1280; Shimadzu, Kyoto, Japan) and the method described by Rinawati et al. (2020) were used. Measurement of carotenoid levels begins with centrifuging 10 mL of samples at a speed of 3000 rpm for 15 min. The supernatant from the centrifugation is discarded, and the samples pellet at the bottom of the tube is extracted with 5 mL of ethanol and 5 mL of diethyl ether. The mixture of methanol and diethyl ether is prepared by adding a NaCl solution. The combined ether fraction is saponified with 2 mL of ethanol‐KOH (final base concentration of 5%) and incubated for 12–16 h at room temperature. Excess base in the filtrate is removed by adding water. The diethyl ether phase is then measured for its absorbance at a wavelength of 452 nm. Carotenoid concentration (μg/mL) is calculated using the formula: Carotenoid (μg/mL) = 3.68 × *A*_452_, where *A* is sample absorbance. For chlorophyll extraction with the same pathway but used acetone as a solvent and heating in a water bath at 40°C for 1 h was applied. The chlorophyll extract is analyzed using UV–Vis spectroscopy at 645 and 663 nm (Rinawati et al. 2020).

### 2.6 Protein and Moisture Analysis

To determine moisture content, the samples were dried in a drying oven (Memmert UF110, Germany) at 105°C until a constant weight was achieved. Crude protein content (N 6.25) was determined by the Kjeldahl method after acid digestion (Konar et al. 2022).

### 2.7 Water Activity

The water activity (*a*_w_) of the samples was determined using a Lab‐Master *a*_w_ (Novasina, Switzerland) as suggested by Konar et al. (2022).

### 2.8 Color Analysis

The color characteristics (L*, a*, b*, C*, and *h*°) of the samples were determined by using a colorimeter (CR‐400; Konica Minolta, Japan) (Konar et al. 2022).

### 2.9 Hygroscopicity

The hygroscopicity behavior of the samples was assessed following the procedure described in Fritzen‐Freire et al. (2012). One gram of encapsulated powder sample was taken in known weight (*m*_i_) of petri dish. The dish was then placed in an airtight glass desiccator with a relative humidity of 75%, obtained by NaCl saturated solution. This assembly was kept constant at a room temperature of 25°C for 7 days. Upon the end of the storage period, the sample (*m*_f_) was weighed, and hygroscopicity was determined by using Equation (6):

(6) $$\text{Hygroscopicity}\;\left(g\;H_{2}O/100\;g\;\text{sample}\right)=\left(\left(m_{f}-m_{i}\right)/m_{i}\right)\times 100$$

### 2.10 Wettability

The static wetting test method (Hammes et al. 2015) was used to determine the wettability time, which evaluates how quickly a dried material spontaneously absorbs water within seconds.

### 2.11 Statistical Analysis

All statistical evaluations were performed using Design Expert software (Stat‐Ease Inc., trial version 13.0, Minneapolis, USA). The software was used to generate mixture models, calculate regression coefficients, and evaluate linear, interaction, and higher‐order effects among wall material components. The significance of each model term was assessed based on *p* values (*α* = 0.05). Model adequacy was further examined through analysis of variance (ANOVA) including *R* ^2^, adjusted *R* ^2^, predicted *R* ^2^, *F*‐values, and lack‐of‐fit tests. Experimental data for physicochemical properties, pigment content, and color parameters were entered in triplicate, and model predictions were compared with experimental values to validate the fitting quality and reliability of the optimized formulation.

## 3 Results

### 3.1 Effects of Wall Materials

To determine the optimum composition of the drying solution, a study plan with 26‐run was performed using Simplex‐Lattice Mixture Design method (Table 1). In the experimental design, encapsulation yield and encapsulation efficiency for total chlorophylls and total carotenoids were used as the response. The final equations for encapsulation yield (Equation 7), encapsulation efficiency of total chlorophylls (Equation 8) and total carotenoids (Equation 9) in terms of wall material components were provided below:

(7) $$\begin{array}{cc} \text{Encapsulation yield}= & +95.37A+96.79B+99.01C+98.06C+98.40E+6.63\mathrm{AB}+3.94\mathrm{AC}2.45\mathrm{AD}+7.31\mathrm{AE}\\ & +4.06\mathrm{BC}+0.4363\mathrm{BD}-4.54\mathrm{BE}-0.0188\mathrm{CD}-3.23\mathrm{CE}-2.29\mathrm{DE}+389.76\mathrm{ABC}\\ & +737.95\mathrm{ABD}-516.52\mathrm{ABE}-237.01\mathrm{ACD}-318.45\mathrm{BCD} \end{array}$$

(8) $$\begin{array}{cc} \text{Encapsulation efficiency},\text{total chlorophylls}= & +457.74A+99.10B+97.90C+94.70D+76.98E+47.43\mathrm{AB}-33.05\mathrm{AC}\\ & -69.39\mathrm{AD}-36.91\mathrm{AE}-16.76\mathrm{BC}-36.34\mathrm{BD}-3.02\mathrm{BE}-24.62\mathrm{CD}\\ & -48.87\mathrm{CE}+27.47\mathrm{DE} \end{array}$$

(9) $$\begin{array}{cc} \text{Encapsulation efficiency},\text{carotenoid}= & +46.61A+57.34B+64.59C+75.72+44.94E+68.10\mathrm{AB}+72.12\mathrm{AC}\\ & +13.53\mathrm{AD}+58.02\mathrm{AE}+138.38\mathrm{BC}+\mathrm{BD}+118.92\mathrm{BE}+103.01\mathrm{CD}\\ & +32.42\mathrm{CE}+133.59\mathrm{DE}+5754.69\mathrm{ABC}+3000.94\mathrm{ABD}-5009.73\mathrm{ABE}\\ & -5140.84\mathrm{ACD}-2768.44\mathrm{BCD} \end{array}$$

whereas A: microalgae biomass (% in feed solution, dm), B: deactivated yeast (% in feed solution, dm), C: aquafaba (% in feed solution, dm), D: inulin (% in feed solution, dm), and E: maltodextrin (% in feed solution, dm).

**FIGURE 1:**
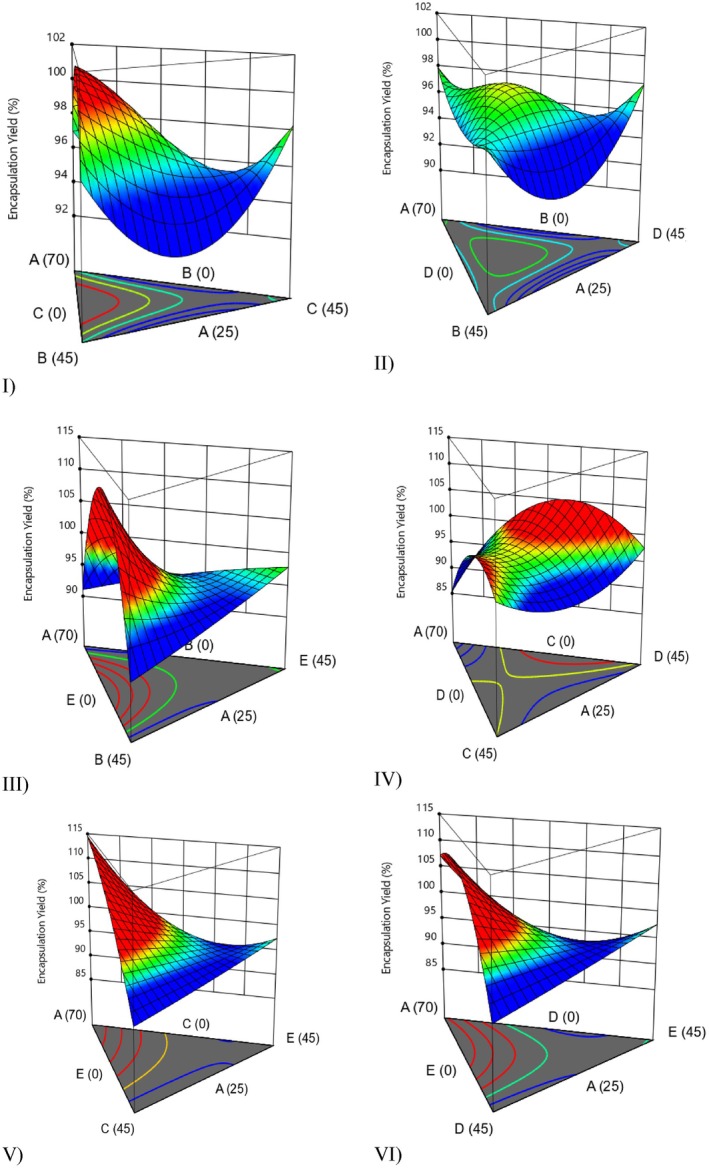
3D surface model graphs illustrating the effect of various wall materials on encapsulation yield (A: Microalgae, B: Deactivated yeast, C: Aquafaba, D: Inulin, and E: Maltodextrin)*. *In all response surface plots, warmer colors (red–yellow) represent higher response values, whereas cooler colors (green–blue) indicate lower values. The curvature of the surface shows how changes in the proportions of the wall materials affect the measured parameter, with upward slopes indicating positive contributions and downward slopes indicating negative effects.

The encapsulation yield ranged from 95.22% to 99.89%. The fitted model for this parameter, with an *R* ^2^ value of 0.9353, was found to be significant (Tamtürk et al. 2023). It was determined that, except for the microalgae‐deactivated yeast‐inulin combination (Figure 1II), all other combinations of materials—microalgae‐deactivated yeast‐aquafaba (Figure 1I), microalgae‐aquafaba‐maltodextrin (Figure 1III), microalgae‐inulin‐maltodextrin (Figure 1IV), microalgae‐deactivated yeast‐maltodextrin (Figure 1V), and microalgae‐aquafaba‐inulin samples (Figure 1VI)—were effective in enhancing encapsulation yield.

**FIGURE 2:**
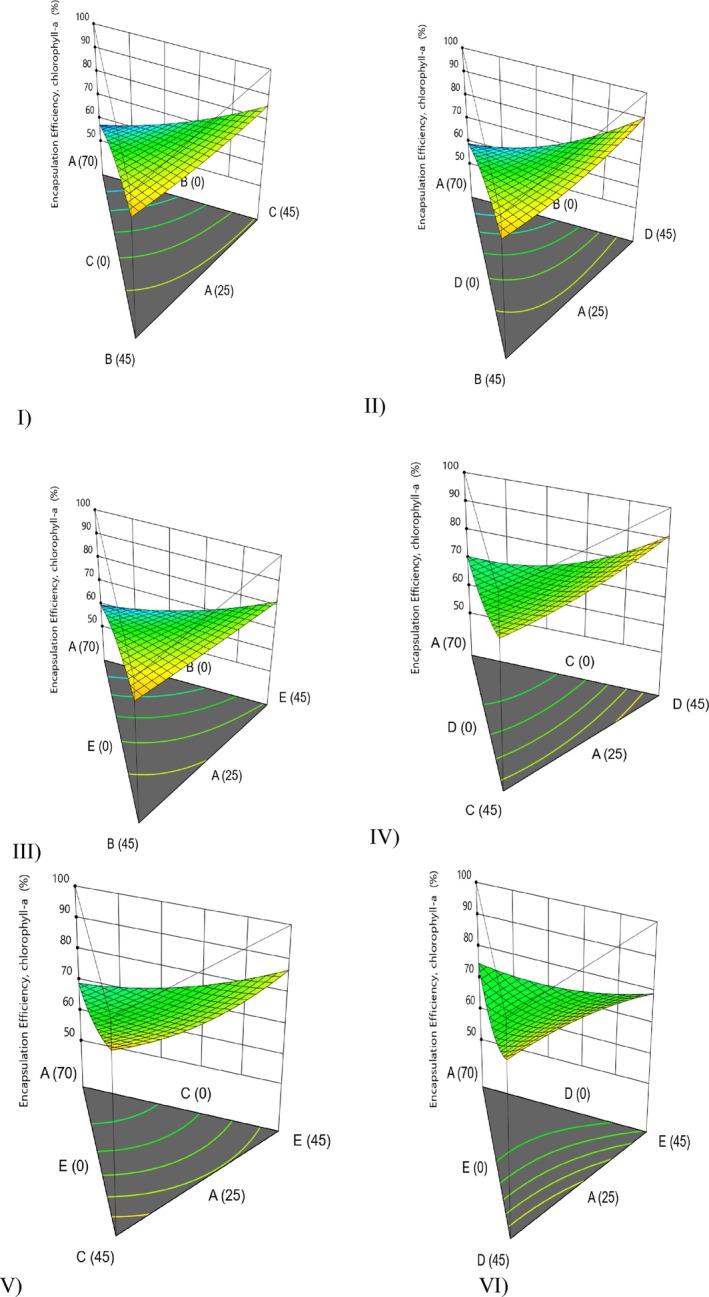
3D surface model graphs illustrating the effect of various wall materials on encapsulation efficiency for chlorophyll‐a (A: Microalgae, B: Deactivated yeast, C: Aquafaba, D: Inulin, and E: Maltodextrin) on encapsulation efficiency for chlorophyll‐a.

When a single‐wall material was used, the encapsulation efficiencies of total chlorophylls and carotenoids ranged from 71.5% to 98.9% and from 41.1% to 81.2%, respectively. When a combination of wall materials was used, these values ranged from 60.5% to 98.9% for total chlorophylls and from 41.1% to 95.6% for total carotenoids. Generally, combining multiple encapsulation agents tended to increase the encapsulation efficiency of total carotenoids, indicating a synergistic interaction. However, this trend was not observed for total chlorophylls. Additionally, the encapsulation efficiency of total chlorophylls decreased with increasing carbohydrate‐based wall material content (Figure 2III,V,VI).

As highlighted in previous sections, *C. vulgaris* biomass is an important protein source. The crude protein content of samples was also determined, ranging from 6.40 ± 0.46 to 43.8 ± 0.36 g/100 g (Table 2). The *R* ^2^ value of the fitted significant linear model was determined as 0.9947 (Table 2). The chemical composition of microalgal biomass depends on factors such as the specific species and strain, growing conditions, and various other factors (Safi et al. 2014).

**TABLE 2:** Physicochemical, wettability and hygroscopicity properties of encapsulated *Chlorella vulgaris* .

Run	Algae biomass (%)	Deactivated yeast (%)	Aquafaba (%)	Inulin (%)	Maltodextrin (%)	pH	Moisture content (g/100 g)	Water activity (*a*_w_)	Crude protein content	Wettability (s)	Hygroscopicity (g/100 g)
1	100	0.00	0.00	0.00	0.00	6.80 ± 0.020	3.02 ± 0.10	0.175 ± 0.004	(g/100 g)	8.50 ± 0.71	11.0 ± 0.47
2	25.0	75.0	0.00	0.00	0.00	5.74 ± 0.010	3.21 ± 0.03	0.164 ± 0.001	29.8 ± 0.68	6.50 ± 0.71	13.3 ± 0.59
3	25.0	0.00	75.0	0.00	0.00	5.18 ± 0.006	2.71 ± 0.05	0.199 ± 0.002	42.4 ± 3.57	6.50 ± 0.71	21.2 ± 0.02
4	25.0	0.00	0.00	75.0	0.00	6.63 ± 0.010	2.29 ± 0.18	0.104 ± 0.001	23.1 ± 0.76	8.50 ± 0.71	11.9 ± 1.01
5	25.0	0.00	0.00	0.00	75.0	6.65 ± 0.006	2.59 ± 0.05	0.132 ± 0.001	7.47 ± 0.62	6.50 ± 0.71	15.3 ± 1.61
6	62.5	37.5	0.00	0.00	0.00	6.20 ± 0.010	2.62 ± 0.08	0.132 ± 0.001	7.32 ± 0.15	13.5 ± 0.71	14.1 ± 0.69
7	62.5	0.00	37.5	0.00	0.00	5.97 ± 0.006	1.63 ± 0.07	0.251 ± 0.028	37.3 ± 1.08	4.00 ± 0.00	14.6 ± 2.49
8	62.5	0.00	0.00	37.5	0.00	6.56 ± 0.012	1.85 ± 0.15	0.107 ± 0.001	27.2 ± 1.27	7.50 ± 0.71	11.6 ± 1.49
9	62.5	0.00	0.00	0.00	37.5	6.38 ± 0.006	1.99 ± 0.07	0.145 ± 0.003	19.2 ± 0.27	8.50 ± 0.71	14.4 ± 1.94
10	25.0	37.5	37.5	0.00	0.00	5.50 ± 0.100	1.30 ± 0.05	0.184 ± 0.001	19.3 ± 0.53	6.50 ± 0.71	18.9 ± 2.28
11	25.0	37.5	0.00	37.5	0.00	5.94 ± 0.012	2.08 ± 0.22	0.115 ± 0.001	35.4 ± 0.18	3.50 ± 0.71	13.1 ± 0.21
12	25.0	37.5	0.00	0.00	37.5	6.33 ± 0.025	1.93 ± 0.10	0.110 ± 0.001	25.4 ± 1.21	3.50 ± 0.71	13.8 ± 0.25
13	25.0	0.00	37.5	37.5	0.00	5.51 ± 0.006	2.31 ± 0.19	0.245 ± 0.001	26.6 ± 0.52	2.50 ± 0.71	14.3 ± 1.42
14	25.0	0.00	37.5	0.00	37.5	5.43 ± 0.010	2.15 ± 0.07	0.197 ± 0.001	14.2 ± 0.08	2.50 ± 0.71	17.8 ± 0.71
15	25.0	0.00	0.00	37.5	37.5	6.63 ± 0.015	1.79 ± 0.07	0.132 ± 0.005	14.5 ± 1.13	5.50 ± 0.71	16.2 ± 1.42
16	70.0	7.50	7.50	7.50	7.50	6.31 ± 0.015	2.55 ± 0.04	0.121 ± 0.003	6.40 ± 0.46	11.5 ± 0.71	10.2 ± 1.23
17	32.5	45.0	7.50	7.50	7.50	5.99 ± 0.006	2.22 ± 0.12	0.126 ± 0.003	25.0 ± 0.72	3.50 ± 0.71	16.4 ± 1.41
18	32.5	7.50	45.0	7.50	7.50	5.52 ± 0.006	2.93 ± 0.05	0.197 ± 0.003	32.9 ± 1.04	2.50 ± 0.71	20.0 ± 0.05
19	32.5	7.50	7.50	45.0	7.50	6.35 ± 0.015	2.94 ± 0.15	0.163 ± 0.002	23.4 ± 0.41	2.50 ± 0.71	16.3 ± 1.55
20	32.5	7.50	7.50	7.50	45.0	6.35 ± 0.010	2.12 ± 0.08	0.139 ± 0.001	15.0 ± 0.28	5.50 ± 0.71	12.4 ± 1.18
21	40.0	15.0	15.0	15.0	15.0	5.94 ± 0.012	3.23 ± 0.14	0.221 ± 0.001	15.4 ± 0.12	4.50 ± 0.71	12.5 ± 1.83
22	100.0	0.00	0.00	0.00	0.00	6.56 ± 0.006	3.36 ± 0.07	0.187 ± 0.001	23.5 ± 0.35	8.50 ± 0.71	9.03 ± 1.35
23	25.0	75.0	0.00	0.00	0.00	5.64 ± 0.015	3.15 ± 0.03	0.238 ± 0.384	30.8 ± 0.33	3.50 ± 0.71	5.06 ± 0.64
24	25.0	0.00	75.0	0.00	0.00	5.13 ± 0.025	2.53 ± 0.04	0.281 ± 0.003	43.8 ± 0.36	2.50 ± 0.71	21.1 ± 2.40
25	25.0	0.00	0.00	75.0	0.00	6.64 ± 0.006	2.98 ± 0.07	0.142 ± 0.006	24.0 ± 0.22	10.50 ± 0.71	16.8 ± 0.86
26	25.0	0.00	0.00	0.00	75.0	6.66 ± 0.010	2.13 ± 0.05	0.135 ± 0.001	8.13 ± 0.22	3.50 ± 0.71	13.1 ± 1.37
Model						Quadratic	S. Quartic	R. Quadratic	8.55 ± 0.08	Quadratic	Linear
*p* value						< 0.0001*	0.0294*	0.02454*	Linear	0.0245*	0.0003*
Lack of fit *F* value						0.8150**	3.9**	0.3967**	< 0.0001*	1.1014**	0.5070**
Model *R* ^2^						0.9898	0.9389	0.5015	1.43**	0.8113	0.6157

*Note:* Mean ± standard deviation. S.: special. Analyses were performed in triplicate.

* Significant.

** Not significant. *p* < 0.05.

The moisture content of the final microalgae samples ranged from 1.30 to 3.23 g/100 g. Among other physico‐chemical properties, the water activity values of the encapsulated microalgae samples were determined to be less than 0.281 ± 0.003, and *R* ^2^ value of 0.5015 for the fitted model (Table 2). Another important stability parameter for powders is hydroscopicity. The total carotenoid content ranged from 0.42 to 1.62 mg/g, whereas the total chlorophyll content varied from 8.51 to 29.84 mg/g (Table 3). The fitted models for total carotenoid and total chlorophyll content exhibited a high goodness of fit, with *R* ^2^ values of 0.9947 and 0.9818, respectively. Moreover, statistical significance was confirmed with very low *p*‐values (< 0.0001). The F value for carotenoids was 6.59, indicating a good fit to the data, whereas for chlorophylls it was 0.2977. Pigment contents have interactions with the visual characteristics of the samples, which are identified using color parameters. The L*, a*, b*, C* and *h*° values of the encapsulated *C. vulgaris* samples were determined within the following ranges: 37.0–57.1 for L*, (−1.27) to (−12.4) for a*, 1.89–23.5 for b*, 2.27–26.2 for C*, and 112.2–125.5 for *h*° (Table 4). The highest extraction yields of carotenoids and chlorophyll were reported as 2.89 mg/g dry biomass and 2.24 µg/mg dry biomass, respectively, using supercritical fluid extraction from Nannochloropsis gaditana (Macıas‐Sánchez et al. 2005) As algae contain significant amounts of bioactive and nutritional compounds (Karimidastjerd et al. 2025) spray drying is considered a promising and encapsulating health‐promoting components such as carotenoids and chlorophyll (Mobasserfar et al. 2026).

**TABLE 3:** Pigment contents of encapsulated *Chlorella vulgaris* .

Run	Algae biomass (%)	Deactivated yeast (%)	Aquafaba (%)	Inulin (%)	Maltodextrin (%)	Chlorophyll‐a (mg/g)	Total carotenoid (mg/g)
1	100	0.00	0.00	0.00	0.00		
2	25.0	75.0	0.00	0.00	0.00	29.8 ± 0.43	1.62 ± 0.49
3	25.0	0.00	75.0	0.00	0.00	11.6 ± 0.33	0.48 ± 0.08
4	25.0	0.00	0.00	75.0	0.00	11.6 ± 0.05	0.53 ± 0.03
5	25.0	0.00	0.00	0.00	75.0	11.5 ± 0.11	0.61 ± 0.11
6	62.5	37.5	0.00	0.00	0.00	9.69 ± 0.19	0.36 ± 0.11
7	62.5	0.00	37.5	0.00	0.00	26.9 ± 0.18	1.49 ± 0.06
8	62.5	0.00	0.00	37.5	0.00	22.6 ± 2.60	1.59 ± 0.07
9	62.5	0.00	0.00	0.00	37.5	18.0 ± 0.01	1.39 ± 0.33
10	25.0	37.5	37.5	0.00	0.00	18.0 ± 0.35	1.30 ± 0.50
11	25.0	37.5	0.00	37.5	0.00	11.3 ± 0.09	0.83 ± 0.07
12	25.0	37.5	0.00	0.00	37.5	10.6 ± 1.10	0.67 ± 0.10
13	25.0	0.00	37.5	37.5	0.00	10.6 ± 1.02	0.70 ± 0.05
14	25.0	0.00	37.5	0.00	37.5	10.9 ± 1.15	0.83 ± 0.20
15	25.0	0.00	0.00	37.5	37.5	9.21 ± 0.61	0.57 ± 0.09
16	70.0	7.50	7.50	7.50	7.50	11.4 ± 0.08	0.81 ± 0.06
17	32.5	45.0	7.50	7.50	7.50	21.0 ± 0.18	1.52 ± 0.04
18	32.5	7.50	45.0	7.50	7.50	14.3 ± 1.01	0.97 ± 0.13
19	32.5	7.50	7.50	45.0	7.50	12.7 ± 0.21	0.83 ± 0.01
20	32.5	7.50	7.50	7.50	45.0	12.1 ± 0.78	0.70 ± 0.02
21	40.0	15.0	15.0	15.0	15.0	10.6 ± 0.29	0.55 ± 0.08
22	100.0	0.00	0.00	0.00	0.00	14.8 ± 1.84	0.71 ± 0.11
23	25.0	75.0	0.00	0.00	0.00	25.2 ± 0.10	1.61 ± 0.22
24	25.0	0.00	75.0	0.00	0.00	11.8 ± 0.92	0.51 ± 0.04
25	25.0	0.00	0.00	75.0	0.00	11.5 ± 3.47	0.59 ± 0.05
26	25.0	0.00	0.00	0.00	75.0	11.3 ± 1.71	0.70 ± 0.03
Model						8.51 ± 0.28	0.42 ± 0.04
*p* value						Quadratic	S. Cubic
Lack of fit *F* value						< 0.0001*	< 0.0001*
Model *R* ^2^						0.2977**	6.59**

*Note:* Mean ± standard deviation. S.: special. Analyses were performed in triplicate.

* Significant.

** Not significant. *p* < 0.05.

**TABLE 4:** Color properties of encapsulated *Chlorella vulgaris* .

Run	Algae biomass (%)	Deactivated yeast (%)	Aquafaba (%)	Inulin (%)	Maltodextrin (%)	a*	b*	L*	C*	*h*°
1	100	0.00	0.00	0.00	0.00	−5.72 ± 0.005	9.38 ± 0.17	49.2 ± 1.15	11.0 ± 0.14	121.4 ± 0.51
2	25.0	75.0	0.00	0.00	0.00	−12.4 ± 0.097	21.2 ± 0.28	54.1 ± 0.68	25.6 ± 1.92	120.4 ± 0.14
3	25.0	0.00	75.0	0.00	0.00	−11.7 ± 0.140	19.5 ± 0.32	52.0 ± 0.56	22.7 ± 0.35	121.0 ± 0.12
4	25.0	0.00	0.00	75.0	0.00	−11.7 ± 0.266	23.5 ± 0.63	57.1 ± 0.86	26.2 ± 0.68	116.5 ± 0.11
5	25.0	0.00	0.00	0.00	75.0	−8.50 ± 0.185	16.1 ± 0.45	48.9 ± 0.99	18.2 ± 0.49	117.8 ± 0.16
6	62.5	37.5	0.00	0.00	0.00	−6.01 ± 0.262	8.43 ± 0.51	40.9 ± 0.66	10.4 ± 0.57	125.5 ± 0.46
7	62.5	0.00	37.5	0.00	0.00	−6.90 ± 0.102	10.4 ± 0.26	42.9 ± 0.22	12.5 ± 0.28	123.7 ± 0.29
8	62.5	0.00	0.00	37.5	0.00	−5.70 ± 0.179	10.6 ± 0.43	47.3 ± 1.72	12.0 ± 0.46	118.3 ± 0.26
9	62.5	0.00	0.00	0.00	37.5	−5.51 ± 0.155	9.92 ± 0.29	42.6 ± 0.47	11.4 ± 0.33	119.0 ± 0.05
10	25.0	37.5	37.5	0.00	0.00	−9.82 ± 0.314	19.5 ± 0.66	52.1 ± 1.11	21.9 ± 0.73	116.7 ± 0.04
11	25.0	37.5	0.00	37.5	0.00	−10.40 ± 0.348	20.1 ± 0.08	53.4 ± 0.28	22.6 ± 0.09	116.9 ± 0.08
12	25.0	37.5	0.00	0.00	37.5	−9.01 ± 0.185	17.7 ± 0.39	55.9 ± 0.58	19.9 ± 0.42	117.0 ± 0.26
13	25.0	0.00	37.5	37.5	0.00	−8.96 ± 0.142	18.2 ± 0.36	52.1 ± 0.54	20.3 ± 0.39	116.2 ± 0.10
14	25.0	0.00	37.5	0.00	37.5	−8.52 ± 0.176	17.5 ± 0.41	50.0 ± 0.59	19.5 ± 0.44	116.0 ± 0.07
15	25.0	0.00	0.00	37.5	37.5	−8.14 ± 0.111	18.2 ± 0.31	51.6 ± 0.70	19.9 ± 0.32	114.1 ± 0.07
16	70.0	7.50	7.50	7.50	7.50	−2.67 ± 0.076	4.19 ± 0.19	38.5 ± 0.04	4.97 ± 0.19	112.2 ± 0.16
17	32.5	45.0	7.50	7.50	7.50	−8.20 ± 0.301	16.1 ± 0.63	48.6 ± 0.61	18.1 ± 0.71	117.0 ± 0.06
18	32.5	7.50	45.0	7.50	7.50	−7.31 ± 0.125	14.2 ± 0.29	46.0 ± 0.27	15.9 ± 0.32	117.3 ± 0.08
19	32.5	7.50	7.50	45.0	7.50	−8.14 ± 0.127	16.5 ± 0.31	49.0 ± 0.36	18.4 ± 0.34	116.3 ± 0.07
20	32.5	7.50	7.50	7.50	45.0	−6.74 ± 0.283	13.7 ± 0.63	47.6 ± 0.67	15.3 ± 0.70	116.1 ± 0.11
21	40.0	15.0	15.0	15.0	15.0	−5.78 ± 0.326	10.8 ± 0.72	43.3 ± 0.98	12.3 ± 0.79	118.1 ± 0.25
22	100.0	0.00	0.00	0.00	0.00	−1.27 ± 0.131	1.89 ± 0.22	37.0 ± 0.26	2.27 ± 0.25	124.0 ± 0.33
23	25.0	75.0	0.00	0.00	0.00	−7.68 ± 0.355	15.8 ± 0.75	55.4 ± 0.89	17.6 ± 0.84	116.0 ± 0.05
24	25.0	0.00	75.0	0.00	0.00	−7.98 ± 0.017	16.6 ± 0.05	49.1 ± 0.07	18.4 ± 0.05	115.7 ± 0.03
25	25.0	0.00	0.00	75.0	0.00	−8.91 ± 0.012	20.6 ± 0.11	56.7 ± 0.05	22.5 ± 0.11	113.4 ± 0.08
26	25.0	0.00	0.00	0.00	75.0	−6.30 ± 0.144	13.9 ± 0.37	47.5 ± 0.78	15.3 ± 0.41	114.3 ± 0.10
Model						Linear	Quadratic	Quadratic	Quadratic	R. Cubic
*p* value						< 0.0001*	0.0022*	0.0085*	0.0071*	0.0040*
Lack of fit *F* value						0.1662**	0.4001**	0.3111**	0.3044**	0.6211**
Model *R* ^2^						0.6751	0.8872	0.8504	0.8562	0.6008

*Note:* Mean ± standard deviation. S.: special. Analyses were performed in triplicate.

* Significant.

** Not significant. *p* < 0.05.

### 3.2 Validation of the Model

Design Expert software was used to generate an optimal wall material composition for the selected parameters to maximize encapsulation efficiencies of total chlorophylls and carotenoids (EE_2_, %) when an encapsulation yield is between 0.0% and 100%. The optimum formulation, which achieved a desirability value of 0.9556, was designed with a feed composition including 61.989% microalgae, 36.989% deactivated yeast, and 1.022% aquafaba in dry mass of feed solution. The experimental values closely matched the predictions made by the model. To validate the models, experimental and predicted values were compared, and the results were found to be within the 95% prediction intervals (Table 5).

**TABLE 5:** Validation results for optimum formulation.^a^

Response	Predicted mean	Experimental data	Accuracy
EY	98.811	97.22 ± 0.31	0.0161
EE_1_	95.56	91.41 ± 0.27	0.0435
EE_2_	89.842	88.46 ± 7.05	0.0154
Moisture content	nd	3.37 ± 0.26	nd
L*	nd	36.62 ± 0.44	nd
a*	nd	−4.17 ± 0.29	nd
b*	nd	4.89 ± 0.14	nd
Chroma	nd	6.35 ± 0.25	nd
Hue angle	nd	131.1 ± 2.20	nd
Wettability	nd	8.50 ± 0.70	nd
Hygroscopicity	nd	1.49 ± 0.08	nd
Water activity	nd	0.25 ± 0.03	nd

*Note:* Mean ± standard deviation.

Abbreviations: EE_1_, chlorophyll‐a encapsulation efficiency; EE_2_, total carotenoids encapsulation efficiency; nd, not determined.

^a^ Feed composition including 61.989% microalgae, 36.989% deactivated yeast, and 1.022% aquafaba in dry mass of feed solution.

## 4 Discussion

### 4.1 Encapsulation Yield and Efficiency

The chemical properties of microalgae undergo changes during drying and encapsulation processes, which can negatively impact their nutritional quality. The content of proteins, lipids, pigments, and other nutrients essential to the food industry may decrease during these processes. Functional components of microalgae, such as pigments, are particularly susceptible to drying conditions including time, temperature, and oxygen exposure (Neves et al. 2019). For this reason, determining optimum wall materials for algae encapsulation and drying is crucial. In this study, we optimized the type and composition of wall material for *C. vulgaris* encapsulation using wall materials such as aquafaba, deactivated yeast, maltodextrin, and inulin for freeze‐drying.

In encapsulation optimization studies, the encapsulation yield is a key parameter for assessing cost efficiency, which is the most important parameter in the industrialization of innovative wall materials. The use of wall materials containing oligo‐ and polysaccharides and proteins may result in higher encapsulation yields during drying and encapsulation of *C. vulgaris* . In a previous study, the encapsulation yields for carotenoids using soy protein as the encapsulating agent were determined to be 96.40% at a 1:1 ratio and 97.46% at a 1:2 ratio for freeze drying (Nogueira et al. 2017).

Microalgal pigments are valuable nutraceuticals and food ingredients known for their potent antioxidant activity. They serve as food supplements and coloring agents, with the biomass of microalgae containing a wide variety of pigments such as carotenoids, chlorophylls, and phycobilins (Fonseca et al. 2020). Chlorophylls are fundamental molecules of life that occur naturally and are possibly the most important and widespread of all natural pigments (Sigurdson et al. 2017). Furthermore, carotenoids are natural pigments found in plants, algae, bacteria, and fungi and are used in foods for both coloring and associated health benefits (Konar et al. 2022). The fitted models for the encapsulation efficiency of total chlorophylls and total carotenoids were found to be significant with *R* ^2^ values of 0.9511 and 0.9723, respectively. The lack of fit for these two parameters was not significant.

Higher encapsulation efficiencies for total carotenoids were also observed in studies in which encapsulation agents with protein content, such as deactivated yeast and aquafaba alone, were used (Table 1). In particular, the use of mixtures of microalgae, deactivated yeast, aquafaba (Figure 2I), and mixtures of microalgae biomass, deactivated yeast, and inulin (Figure 2II) resulted in the highest efficiencies both for total chlorophylls and also total carotenoids (Table 1). For microalgae‐aquafaba‐inulin (Figure 2IV), maximum chlorophyll‐a encapsulation efficiency at 25%‐45%‐45% line was observed. Notably, using deactivated yeast has great potential for microalgae encapsulation, considering the sustainable supply of this material (Tamtürk et al. 2023). Chemical pretreatment of yeast cells to deplete the cell contents can improve the capacity to take up core materials and ultimately enable better encapsulation efficiency (Dadkhodazade et al. 2021; Nguyen et al. 2018). The composition of the yeast cell wall and plasma membrane significantly impacts how effective the encapsulation process is. The combined effects of proteins, polysaccharides, lipids, and nucleic acids within yeast cells profoundly affect their encapsulation properties (Shi et al. 2010). This process increases the intracellular spaces and thus improves the encapsulation capacity of the carriers (Dadkhodazade et al. 2021). Pretreatment approaches for yeast cells include various applications that can affect encapsulation efficiency differently (Czerniak et al. 2015).

The encapsulation efficiency of total chlorophylls was affected, and it decreased with increasing carbohydrate wall material content. Agarry et al. (2022) used soy protein isolate and chitosan as carriers to encapsulate chlorophyll. They highlighted that soy protein isolate provides more binding sites for chlorophyll, which leads to enhanced interactions between soy protein isolate and chlorophyll, increasing encapsulation efficiency, according to their research results. Also, in previous studies using soy protein and freeze‐drying, the encapsulation efficiency of total carotenoids was determined as nearly 65.0% (Nogueira et al. 2017) and 42.89% (Stramarkou et al. 2017).

### 4.2 Physicochemical Properties

The pH values of encapsulated *C. vulgaris* powders ranged from 5.13 to 6.80, reflecting a slightly acidic to near‐neutral environment. Formulations with higher proportions of algae biomass alone tended to have slightly higher pH values (~6.5–6.8), whereas aquafaba‐ or yeast‐rich formulations were more acidic (pH 5.1–5.9), likely due to the intrinsic acidity of these wall materials. These pH variations can influence powder stability, solubility, and compatibility with food matrices. Final moisture contents varied from 1.30 to 3.36 g/100 g, demonstrating the effectiveness of freeze‐drying in producing low‐moisture powders. Runs with higher aquafaba or yeast content generally had slightly higher moisture, possibly due to their hydrophilic nature, whereas algae‐ or maltodextrin‐rich powders exhibited lower moisture levels, enhancing shelf stability. Water activity values were very low, ranging from 0.104 to 0.281, which is favorable for long‐term storage. Powders with higher algae biomass content showed lower water activity, whereas aquafaba‐containing samples had higher aw, reflecting their greater moisture retention capacity. Water activity below 0.3 ensures limited microbial growth and slows down chemical or enzymatic reactions, confirming the suitability of these powders for safe, extended shelf life. The protein values obtained here align well with previously reported data for Chlorella strains (Safi et al. 2014). Despite this naturally high protein content of the biomass, the final crude protein concentration of encapsulated powders depends strongly on the wall materials used. Aquafaba formulations typically contain 18–26 g/100 g protein, depending on chickpea cultivar and processing conditions (Buhl et al. 2019; Shim et al. 2018). Yeast cells and yeast‐derived ingredients contain considerably higher protein levels (> 45%) and are often used to enhance nutritional quality in food matrices (Pacheco et al. 1997; Ahiwe et al. 2021). In contrast, inulin and maltodextrin—both carbohydrate‐based encapsulants—contain negligible protein. Consequently, in the present work, the crude protein content of powders varied widely, reflecting the proportion of *C. vulgaris* biomass relative to protein‐rich (yeast, aquafaba) or protein‐poor (inulin, maltodextrin) carriers. These values fall within the expected range for microalgal products and align with previously published data for Chlorella‐based formulations (Safi et al. 2014; Konar et al. 2022).

Wettability values among the formulations ranged widely (2.50–13.5 s), indicating that the type and proportion of wall materials significantly influenced the rehydration behavior of the freeze‐dried powders. Lower wettability times reflect faster dispersion and improved surface hydrophilicity.

Formulations containing aquafaba and inulin (Runs 7, 10, 13, 14, 17, 18) exhibited the fastest wetting, with values as low as 2.50 s, suggesting that these components enhanced the powder's ability to absorb water. Aquafaba contains surface‐active proteins and saponins, whereas inulin provides soluble dietary fibers; both contribute to rapid water penetration into the particle matrix. These findings align with previous reports indicating that plant‐based foaming agents and soluble fibers can improve wettability by increasing porosity and reducing surface tension at the powder–water interface. In contrast, formulations with high proportions of maltodextrin or pure algal biomass (Runs 1, 5, 9, 15) showed slower wetting (up to 13.5 s). Maltodextrin's dense microstructure and lower surface activity reduce the number of hydrophilic sites available for water interaction, whereas pure Chlorella biomass contains rigid cell walls that inherently limit water penetration. Similar delays have been reported in microalgal powders encapsulated without strong surfactants or foaming agents. Overall, the results show that aquafaba and inulin improved the rehydration properties, whereas excessive maltodextrin or unencapsulated algae decreased wettability.

Hygroscopicity values varied between 5.06 and 21.2 g/100 g, reflecting differences in moisture‐binding capacity among formulations. Higher hygroscopicity indicates greater affinity for atmospheric moisture, which can affect storage stability and caking tendencies. The highest hygroscopicity values were observed in formulations containing high aquafaba or inulin proportions (e.g., Runs 3, 7, 13, 18, 24), reaching up to 20–21 g/100 g. Both aquafaba and inulin are rich in hydrophilic functional groups (–OH, –COOH), which readily bind water molecules. Inulin's known prebiotic fiber structure contributes to strong water sorption, whereas proteins and polysaccharides in aquafaba increase surface hydrophilicity. This pattern is consistent with previous research demonstrating that soluble carbohydrates and protein‐rich binders increase moisture uptake in encapsulated powders. Lower hygroscopicity was recorded in formulations dominated by maltodextrin or algal biomass (e.g., Runs 1, 5, 9, 20, 23), with values ranging from 5.06–14 g/100 g. Maltodextrin, especially at higher DE levels, exhibits moderate hygroscopicity due to its shorter chains, but its glassy structure limits extensive water absorption. Similarly, pure Chlorella biomass contains more lipids and rigid cell components, reducing its moisture‐binding capacity. Thus, hygroscopicity was largely dictated by the type of wall material, where more hydrophilic carriers (aquafaba, inulin) produced powders with higher moisture affinity, whereas maltodextrin and algal biomass resulted in more storage‐stable, less hygroscopic powders.

In the food and feed industry, freeze‐drying is widely used as a commercial drying method to obtain high‐quality microalgae biomass. However, its high capital and operating costs and long drying time are the primary drawbacks of this method (Kim and Kim 2022). The main purpose of encapsulation with the freeze‐drying method is to reduce the moisture levels while minimizing damage to bioactive components, thereby limiting microbial growth and oxidative reactions. Stramarkou et al. (2017) while minimizing damage to bioactive components, thereby limiting microbial growth and oxidative reactions. Similarly, a previous study dried marine *Chlorella* sp. biomass with an initial moisture content of 90 g/100 g at −50°C/12.66 Pa for 24 h, yielding a final moisture content of 8.10 g/100 g (Amin et al. 2021). Typically, a target moisture content of 5.00 g/100 g is recommended for drying microalgae (Kim and Kim 2022). The moisture content of food products affects shelf life and various quality properties. However, water activity (*a*_w_) is a curcial parameter for determining biochemical reactions and microbiological stability (Vimercati et al. 2022). The water activity values of the encapsulated microalgae samples were lower than 0.3. For powdered products, the water activity less than 0.3 limits the growth of most microorganisms and the occurrence of chemical and enzymatic reactions (Konar et al. 2022). Another parameter that affects the stability of powder products is hygroscopicity, that is, the ability to absorb moisture (Oliveira et al. 2022; Todorović et al. 2022; Fritzen‐Freire et al. 2012). Particularly, the samples encapsulated with aquafaba had higher hygroscopicity than others. In a previous study, the hygroscopicity for encapsulated coffee silverskin extracts was determined to be 18.2 g/100 g using freeze‐drying (Vimercati et al. 2022). The high hygroscopicity of aquafaba is attributed to its branched structure, similar to amylopectin, which facilitates hydrogen bonding with water molecules (Kim and Shin 2022). The combination of low moisture content and water activity, along with a slightly acidic to neutral pH, indicates that the encapsulated powders are well‐suited for stable storage. Variations are clearly dependent on the type and proportion of wall materials, highlighting the importance of formulation in powder quality and stability.

### 4.3 Pigment Content

*Chlorella vulgaris* potentially contains 1.002.00 g/100 g of chlorophyll (Safi et al. 2014), as well as approximately 0.4 g/100 g carotenoids (e.g., astaxanthin, lutein, β‐carotene, lycopene, and canthaxanthin) (Rua et al. 2020). Based on results, significant models with relatively high *R* ^2^ values were determined for effects of wall materials on pigment contents. Feed solutions containing *C. vulgaris* (%25, dry basis) and deactivated yeast, aquafaba or inulin, were found to have higher total chlorophylls and carotenoid amounts than samples prepared with maltodextrin. Significant increases in total carotenoid contents were observed in samples containing two different wall materials, which may be explained by their synergistic effect. Chlorophyll‐a, the primary green pigment in *C. vulgaris* , ranged widely from 9.21 to 29.8 mg/g across the formulations. The highest chlorophyll‐a content was observed in formulations with low algae content but high yeast or aquafaba proportion (e.g., Run 2: 25% algae +75% yeast, 29.8 mg/g; Run 7: 62.5% algae +37.5% aquafaba, 26.9 mg/g). This suggests that certain wall materials may protect or enhance pigment retention during encapsulation and freeze‐drying. Pure algae samples (Runs 1, 22, 100% algae) had moderately high chlorophyll‐a values (14.8 mg/g for Run 22), but some reduction may occur due to oxidative degradation during drying, especially without protective wall materials. Formulations with inulin or maltodextrin as the main wall material generally showed lower chlorophyll‐a (≈9–12 mg/g), indicating these carriers may be less effective at preserving chlorophyll during processing.

Total carotenoids ranged from 0.36 to 1.62 mg/g, with patterns similar to chlorophyll‐a. The highest carotenoid content was observed in formulations with yeast or aquafaba as wall materials (Run 2: 1.62 mg/g; Run 7: 1.49 mg/g), suggesting protective effects against oxidation. Formulations dominated by inulin or maltodextrin exhibited lower carotenoid retention (≈0.5–0.7 mg/g), again reflecting lower protective capacity of these wall materials for sensitive pigments. Yeast and aquafaba appear to enhance pigment stability, likely due to their protein content and structural properties that provide a physical barrier against light, oxygen, and heat during freeze‐drying. In contrast, carbohydrate‐rich matrices like inulin and maltodextrin offer less protection, resulting in reduced pigment retention. The data indicate that pigment preservation is strongly dependent on wall material composition, rather than algae concentration alone. The model for chlorophyll‐a was quadratic (*p* < 0.0001), indicating significant interaction effects between algae content and wall materials. Total carotenoid content followed a cubic model (*p* < 0.0001), suggesting complex interactions in how wall materials influence carotenoid stability. The R^2^ values indicate moderate model fit, meaning other unmeasured factors (e.g., drying parameters, particle size) may also affect pigment retention.

These observations align well with previous studies. For instance, a recent investigation of microencapsulated chlorophyll using a blend of polysaccharide (maltodextrin) and protein (whey protein isolate, WPI) found that the carrier type significantly impacted chlorophyll stability and retention—soy or pea protein isolates combined with inulin provided superior chlorophyll retention and antioxidant capacity compared with polysaccharide‐only carriers (Turkiewicz et al. 2025). Similarly, when encapsulating β‐carotene in a pullulan (polysaccharide) + WPI (protein) matrix, both spray‐drying and freeze‐drying produced encapsulates with reasonably high encapsulation efficiencies, demonstrating that protein–polysaccharide matrices are effective for carotenoid stabilization (Drosou and Krokida 2024). Other study that used protein–polysaccharide or protein–starch blends (e.g., gelatin + starch) also reported enhanced pigment (or bioactive compound) retention compared with polysaccharide‐only walls, supporting the idea that amphiphilic proteins provide binding sites for hydrophobic pigments, and polysaccharides contribute structural stability (Oladeji and Awolu 2025). In line with these findings, our results suggest that optimizing the mixture of wall materials is essential to maximize pigment retention, because different carriers complement each other: proteins facilitate pigment binding (especially for hydrophobic carotenoids), whereas polysaccharides or carbohydrate‐based materials provide a stable matrix that helps preserve the pigment during freeze‐drying and storage.

### 4.4 Color

It has been reported that synthetic food coloring agents may cause various health problems after consumption, so using natural ones is increasing worldwide (Konar et al. 2022). Some of these ingredients also have various potential health effects that support consumer health and well‐being. The color properties of the samples were evaluated to assess the potential of *C. vulgaris* as a natural pigment source and to characterize their visual attributes. The a* values were negative for all formulations, ranging from −12.4 to −1.27, indicating that all powders exhibited a greenish hue. Formulations with higher yeast or aquafaba content (Runs 2 and 3) showed more negative a* values, that is, stronger green intensity, likely due to color contribution from the wall material interacting with chlorophylls. Conversely, algae‐only powders (Runs 1 and 22) exhibited less negative a* values, suggesting a lighter green. The b* values ranged from 1.89 to 23.5, showing varying yellow tones in the powders. Formulations with higher inulin or maltodextrin content (Runs 4, 5, 25) had higher b* values, indicating more pronounced yellow shades. This may be associated with lower chlorophyll masking or the natural yellowish tint of the wall materials. Lightness values varied widely from 37.0 to 57.1, demonstrating that increasing non‐algal wall materials, particularly maltodextrin and inulin, led to brighter powders. Pure algae powders were darker (lower L*), consistent with their high pigment content. Chroma values, which represent color intensity, ranged from 2.27 to 26.2. Higher chroma values were observed in powders containing more wall materials such as aquafaba and inulin, reflecting more saturated colors, whereas algae‐rich powders had lower chroma due to the dominance of chlorophyll green over other hues. Hue angles ranged from 112.2° to 125.5°, confirming a green‐yellow hue for all formulations. Minor variations in *h*° were linked to differences in wall material composition, with more algae‐rich formulations tending towards greener tones (higher *h*°), and higher maltodextrin/inulin content slightly shifting hue towards yellow. Samples prepared using inulin (%75) had higher L*, b*, C* values. Using deactivated yeast (%75) resulted with higher a* values. These findings were not in accordance with the samples' pigment (total chlorophylls and carotenoids) concentrations. For instance, Stramarkou et al. (2017) observed that maltodextrin‐encapsulated *Chlorella* powders displayed higher lightness, whereas protein‐based carriers induced slight green or red hues depending on the protein type. Similarly, Rubio et al. (2020) found that yeast‐based encapsulation of bioactive extracts altered surface color properties, even when pigment content was preserved. Therefore, colorimetric analysis may be used to investigate visual characteristics. Still, pigment concentrations and their stability during storage and food processes should be determined by considering the specific food application. By colorimetric analysis, the surface properties of powders can be determined. For more efficient encapsulation, these pigments and the entire biomass should be covered by wall materials. Otherwise, the pigments could be exposed to harsh environmental conditions such as oxygen and light. As a result, their stability will be impaired.

### 4.5 Encapsulation Material Selection

The term “aquafaba” refers to the soluble phase of chickpeas that is released into boiling water when cooked (Alsalman and Ramaswamy 2021). It consists primarily of water (92%–95%) and dry matter (5%–8%), which includes carbohydrates (such as sugars, soluble and insoluble fiber), low molecular weight proteins (about 0.95%–1.5% and typically below 24 kDa), saponins and some Maillard reaction products. As a plant‐derived functional ingredient, aquafaba exhibits foaming, emulsifying and gelling properties, making it valuable for the development of novel food products (He et al. 2021). Although extensive research has been conducted on the use of legume proteins as a wall material (Sridhar et al. 2022; Gharibzahedi and Alavinia 2017; Li et al. 2019), only one study has investigated aquafaba‐based encapsulation and/or drying of microalgae (Tamtürk et al. 2023). Aghdam et al. (2021) used isolated chickpea protein for the encapsulation of licorice root extract in their study. Sensory evaluations of model beverages containing microparticles loaded with licorice extract indicated that microencapsulation effectively masked its bitter aftertaste and color.

**FIGURE 3:**
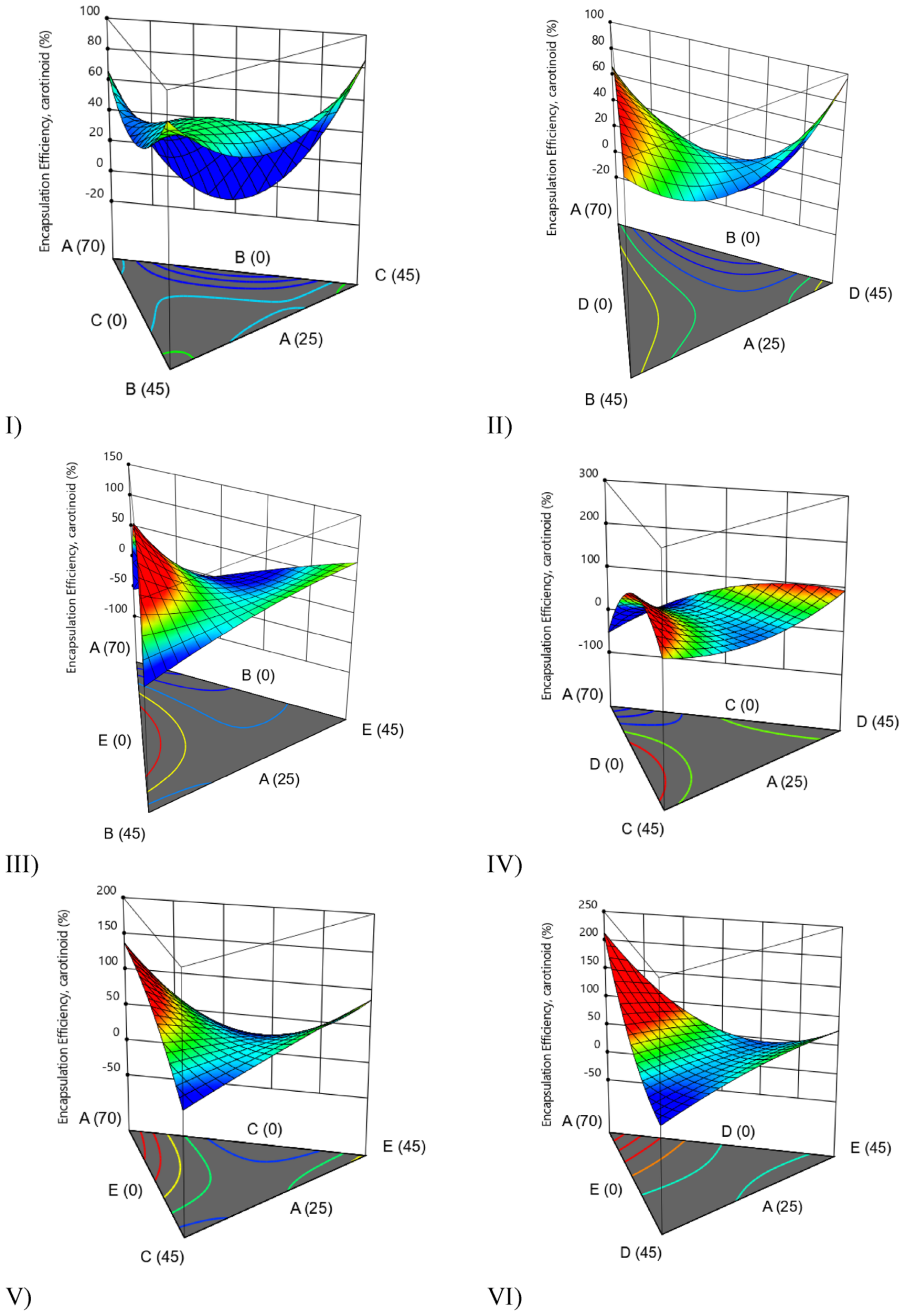
3D surface model graphs illustrating the effect of various wall materials on encapsulation efficiency for total carotenoids (A: Microalgae, B: Deactivated yeast, C: Aquafaba, D: Inulin, and E: Maltodextrin).

Recently, one of the most preferred encapsulation strategies has been the use of yeast cells, as they naturally possess a complex structure and contain both a multilayered cell wall and various intracellular components with high antioxidant capacity. Several studies have been conducted to determine the potential of *S. cerevisiae* yeast cell as a carrier agent for encapsulation of some bioactive compounds and extracts (Karaman 2020). In a previous study, the feasibility of obtaining grape pomace extract powders with low water activity (< 0.289), hygroscopicity (< 13.71 g/100 g) and moisture content (7.10) using these novel materials was investigated. It was also found that the bioaccessibility of grape pomeace polyphenols increased because of using this material (Rubio et al. 2020). Yeast‐based wall materials have been utilized as a natural coating agent for the encapsulation of various food ingredients, effectively protecting both water‐soluble and insoluble molecules such as fish oil, essential oils, chlorogenic acid, curcumin, and enzymes (Sultana et al. 2017). According to our results, yeast‐based encapsulation also can be used for drying and encapsulation of microalgae (Figure 3II–VI). However, microalgae‐deactivated yeast‐maltodextrin combination (Figure 3I) has the lowest total carotenoids.

This study demonstrated the effectiveness of aquafaba and deactivated yeast as innovative wall materials for freeze‐dried *C. vulgaris* . However, real‐food application trials and sensory evaluations were not included, and the investigation was limited to a single microalgal species, thereby restricting the broader applicability of the results. In the future, a wider range of wall material combinations, evaluation of alternative drying technologies, and extended storage studies to assess pigment stability and product shelf life should be explored. In addition, advanced structural characterization and assessments of antioxidant capacity, bioaccessibility, and digestive stability could offer a more comprehensive understanding of its functionality.

## 5 Conclusion

This study successfully demonstrated the potential of innovative wall materials—deactivated yeast and aquafaba—in enhancing the encapsulation and freeze‐drying efficiency of *C. vulgaris* pigments. Encapsulation efficiency models for total chlorophylls, carotenoids, and yield were statistically significant (*p* < 0.05) with high reliability (*R* ^2^ = 0.93–0.97). The validation process confirmed that the optimal formulation achieved superior encapsulation performance. The resulting microalgal powder exhibited desirable physicochemical characteristics that support enhanced storage stability. Its color attributes remained consistent with the natural appearance of *C. vulgaris* , indicating suitability for natural pigment applications. In addition, the solubility behavior of the powder favors its incorporation into a variety of functional food formulations. Overall, this study presents a novel and effective approach for encapsulating *C. vulgaris* using functional and sustainable wall materials, offering promising applications in the development of multi‐functional, stable, and consumer‐preferred food ingredients.

## Author Contributions

**Ömer Said Toker:** conceptualization, writing – review and editing. **Faruk Tamtürk:** methodology, formal analysis. **Başak Gürbüz:** methodology, formal analysis. **Atefeh Karimidastjerd:** writing – review and editing. **Yaşar Durmaz:** conceptualization, writing – review and editing. **Nevzat Konar:** conceptualization, writing – review and editing. **Sevim Dalabasmaz:** writing – review and editing.

## Funding

The authors have nothing to report.

## Conflicts of Interest

The authors declare no conflicts of interest.

## Data Availability

The data that support the findings of this study are available from the corresponding author upon reasonable request.
